# Clinics

**Published:** 1875-01

**Authors:** 


### Clinics.

### COOK COUNTY HOSPITAL.

*Clinics for November. Surgical Department. Service of* PROF. E. POWELL.

(Reported by F. C. WINSLOW, M.D., Assistant Physician.)

I. Stricture of the rectum.
II. Use of the warm bath.
III. Degeneration of the tibia.
IV. Caries of the astragalus.
V. Keloid tumors.
VI. Contusion of the genital organs—Retention of urine—Cystotomy.

I. *Stricture of Rectum.* The patient is a woman about twenty-five years old. She has had trouble in the rectum ever since she can remember. Has been troubled more or less with diarrhœa, the fæces being passed in small quantities and with difficulty. Examination reveals a stricture of the rectum two inches and a half above the anus through which it is impossible to pass the tip of the index finger. The stricture can be seen plainly with the aid of a Sims' duck-bill speculum, which instrument should always be used for inspection of the bowel. Malignancy is excluded, from the fact that a stricture due to the presence of scirrhus would run its course more rapidly, and also from the absence of constitutional symptoms.

The treatment will be conducted upon the plan of dilatation, commencing with a No. 13 flexible urethral bougie and continued by the gradual substitution of larger sizes.

II. *Use of the Warm Bath.* In two cases where symptoms of hospitalism exhibited themselves following slight operations, the following course was pursued: The patient being at the time in a state of nervous irritation, tossing on the bed and complaining of pain, with a pulse 110 per minute and a temperature of 101 degrees, was stripped and placed in an alkaline bath as hot as could be borne, and at the same time potass. brom. gr. xl. was administered. He was permitted to remain in the bath twenty minutes, when he was placed in bed and warmly covered with blankets. Profuse sweating followed the treatment. The temperature came down to 98 degrees, and the patient sank into a refreshing sleep, lasting several hours.

The treatment was repeated every few days as it became necessary.

III. *Degeneration of the Tibia.* The patient is a young woman who received a slight injury upon the leg, six years ago. At present, the anterior aspect of the leg is the seat of a large ulcer, the surface of which is covered with loose, flabby granulations. The tibia was hypertrophied, and consisted of merely a shell of bony tissue upon the outside—the centre of the bone being occupied by a collection of fat and fibrous tissue, soft, friable, and easily scooped out with the "spud." Amputation was advised, but the patient refused to submit to this operation.

IV. *Caries of Astragalus.* The right ankle was the seat of numerous small openings communicating with the bone. The limb has been affected for over a year, commencing with pain and swelling, but without any injury having been received.

The patient is anæsthetized and a semicircular flap of integument raised from the internal aspect of the ankle. Examination showed the astragalus in a carious condition, the entire centre of the bone being softened, leaving only a thin shell of bone. It was decided to amputate the leg at the junction of the lower and middle third. The subsequent progress has been good.

V. *Keloid Tumors.* The affection exists in a negro about forty years of age. He has had the tumors over thirty years, and they are of all sizes, and located all over the neck, trunk, arms and legs. They are not painful, the principal inconvenience being an itching and burning sensation, prompting to constant scratching. One or two of the larger ones have taken on suppurative action, discharging a thin ill-smelling fluid. He is pretty well nourished, has a good appetite and sleeps well. Says that a good deal of his life has been spent in hard labor, but for the last few years he has not done much but try to cure himself. To this end he has visited watering places, bathing and drinking the waters, but has so far received no benefit.

In this case, excision is not practicable, as it would be necessary to skin the man in order to remove them all. He states that one or two have been removed, but quickly reappeared in the same place. He takes a warm bath daily, for the relief of the irritation of the skin, and beyond this nothing is being done for him yet.

VI. *Contusion of the Genital Organs—Retention of Urine—Cystotomy.* The patient was caught between the tiller of a canal boat and the side of the cabin, and suffered severe contusion of the penis and testicles. The accident occurred at 9 P. M., and the patient was brought in the following morning.

He had not passed urine since some time in the afternoon before the injury was received. He was in great distress, and examination revealed the fact that the penis, scrotum and perineum were infiltrated with urine. The operation of perineal section was performed, and the bladder emptied. On regaining consciousness the patient expressed the great relief he experienced as compared to his previous condition. Several free incisions were made in the penis, through which the infiltrated urine might escape. Owing to some tumefaction of the wound the urine was drawn for a few days by means of a flexible catheter introduced through the opening in the perineum. The case progressed without a single untoward symptom.

